# Modulation of Hypercholesterolemia-Induced Oxidative/Nitrative Stress in the Heart

**DOI:** 10.1155/2016/3863726

**Published:** 2015-12-14

**Authors:** Csaba Csonka, Márta Sárközy, Márton Pipicz, László Dux, Tamás Csont

**Affiliations:** Department of Biochemistry, Faculty of Medicine, University of Szeged, Dóm tér 9, Szeged 6720, Hungary

## Abstract

Hypercholesterolemia is a frequent metabolic disorder associated with increased risk for cardiovascular morbidity and mortality. In addition to its well-known proatherogenic effect, hypercholesterolemia may exert direct effects on the myocardium resulting in contractile dysfunction, aggravated ischemia/reperfusion injury, and diminished stress adaptation. Both preclinical and clinical studies suggested that elevated oxidative and/or nitrative stress plays a key role in cardiac complications induced by hypercholesterolemia. Therefore, modulation of hypercholesterolemia-induced myocardial oxidative/nitrative stress is a feasible approach to prevent or treat deleterious cardiac consequences. In this review, we discuss the effects of various pharmaceuticals, nutraceuticals, some novel potential pharmacological approaches, and physical exercise on hypercholesterolemia-induced oxidative/nitrative stress and subsequent cardiac dysfunction as well as impaired ischemic stress adaptation of the heart in hypercholesterolemia.

## 1. Introduction

**Figure 1:**
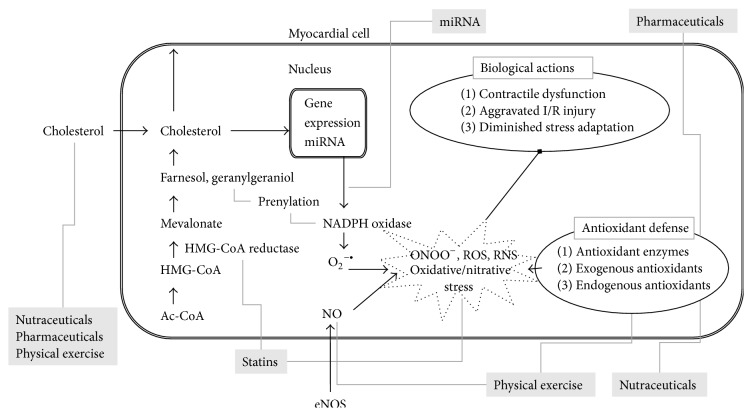
Hypercholesterolemia-induced myocardial oxidative/nitrative stress and its possible modulations (in grey boxes) to prevent or treat deleterious cardiac consequences. Ac-CoA: acetyl-coenzyme A; HMG-CoA: 3-hydroxy-3-methyl glutaryl coenzyme A; eNOS: endothelial nitric oxide synthase; ONOO^−^: peroxynitrite; ROS: reactive oxygen species; RNS: reactive nitrogen species; miRNA: microRNA.

Hypercholesterolemia, a frequent form of hyperlipidemia, is a metabolic disorder characterized by elevated levels of total cholesterol in the blood. Hypercholesterolemia may develop as a consequence of unbalanced diet, obesity, inherited (genetic) diseases (familial hypercholesterolemia), or other diseases (e.g., diabetes). According to large clinical studies, hypercholesterolemia affects a significant population of adults in developed countries [1]. For instance, approximately 100 million people (44.4%) suffered from hypercholesterolemia (>5.2 mmol/L) in the United States in 2008 [2]. The relationship between hypercholesterolemia and cardiovascular mortality has been known for decades [3]. Hypercholesterolemia, especially elevated low density lipoprotein (LDL) cholesterol, is a major risk factor for the development of atherosclerosis and subsequent ischemic heart disease [4], which is a leading cause of death worldwide [5]. Moreover, several experimental studies have demonstrated that, in addition to its well-known proatherogenic effect in the vasculature, hypercholesterolemia may directly affect the heart causing contractile dysfunction [6–8], aggravated ischemia/reperfusion injury [9], and attenuated responses to cardioprotective interventions including ischemic pre- and postconditioning [10, 11]. Although the pathoetiology of hypercholesterolemia has been studied extensively, the precise molecular mechanisms leading to cardiac complications are not entirely clear. Nevertheless, substantial evidence exists demonstrating that hypercholesterolemia induces oxidative and nitrative stress in the heart and that oxidative/nitrative stress plays a role in several cardiac pathologies. Therefore, modulation of oxidative stress in the hypercholesterolemic myocardium appears to be a rational approach. In this review we aim to discuss relevant literature related to potential modulation of hypercholesterolemia-induced oxidative stress and subsequent complications in the heart (Figure 1). Our attention is focused on certain pharmaceuticals, nutraceuticals, novel pharmacological approaches, and physical exercise as potential modulators.

## 2. Hypercholesterolemia and Oxidative/Nitrative Stress

Oxidative/nitrative stress can be defined as an excess formation or insufficient removal of highly reactive molecules such as reactive oxygen and/or nitrogen species (ROS and RNS, resp.) including, for instance, superoxide, hydrogen peroxide, hydroxyl radical, and peroxynitrite [12]. Enzymatic sources for ROS formation include the mitochondrial respiratory chain, nicotinamide adenine dinucleotide phosphate (NADPH) oxidases, xanthine oxidase, cyclooxygenases, uncoupled nitric oxide synthase (NOS), and peroxidases, while antioxidant enzymatic systems include superoxide dismutase (SOD), catalase, glutathione peroxidase, glutathione reductase, and heme oxygenase (HO) [13]. Although there is a consensus in the literature that hypercholesterolemia is associated with increased cardiac oxidative stress (Figure 1), the precise molecular mechanisms by which hypercholesterolemia induces oxidative stress in the heart are not entirely clear. Accumulating evidence shows increased superoxide production in the hearts of hypercholesterolemic animals, and one of the major sources of this superoxide appears to be increased NADPH oxidase activity in cholesterol-fed Wistar rats and apoB100 transgenic mice [6, 8]. In the hearts of cholesterol-fed CFLP mice and Wistar rats, transcript levels of NADPH oxidase 4 (NOX4) were significantly increased when compared to normal controls fed a standard chow [8, 14]. The increased NOX4 transcript levels in hypercholesterolemic hearts can be related to transcriptional, posttranscriptional, or epigenetic regulation of NOX4 expression. One study shows an association of cardiac NOX4 transcript and protein levels as well as NADPH oxidase activity with decreased myocardial level of the microRNA miR-25 in rats with diet-induced hypercholesterolemia [8]. MicroRNAs (miR or miRNA) are endogenous, small, noncoding RNAs that are responsible for posttranscriptional silencing of a wide variety of specific target genes. NOX4 was identified as a direct target of miR-25, suggesting that decreased miR-25 allows the upregulation of NOX4, which contributes to increased ROS production in the hypercholesterolemic heart [8]. Although alterations in cardiac gene expression have been demonstrated in animal models of diet-induced hypercholesterolemia [14, 15] and metabolic syndrome [16], to the best of our knowledge, the precise molecular link between hypercholesterolemia and altered cardiac gene expression has not yet been elucidated. Interestingly, two key transcriptional factors have been implicated to date in cholesterol-dependent transcriptional regulation of gene expression, that is, the sterol-regulatory element binding protein (SREBP) and the liver X receptor (LXR) [17]; however, direct evidence for their role in hypercholesterolemia-induced oxidative stress is still lacking.

Another possible explanation for increased oxidative stress could be diminished endogenous antioxidant capacity. Indeed, hypercholesterolemia has been reported to be associated with decreased cardiac expression and activity of antioxidant enzymes [18–21]. Nevertheless, the precise molecular mechanisms by which high cholesterol downregulates myocardial antioxidant enzymes remain to be elucidated. Besides enzymatic mechanisms, tissue level of the endogenous antioxidants is also an important determinant of oxidative/nitrative stress. Nitric oxide (NO) is a key antioxidant molecule that is synthesized by NO synthases in various cells including cardiomyocytes. In the hearts of hypercholesterolemic animals, cardiac NO level was decreased when compared to controls [10, 22, 23]; however, this effect was independent of the modulatory effect of cholesterol on the mevalonate pathway [23]. Moreover, transcript levels of NOS 3 were decreased, while NOS 1 and 2 remained unaffected in the hearts of cholesterol-fed mice [14].

In addition to the effects of cholesterol and its derivatives on gene expression, membrane-related effects of cholesterol are also plausible [39]; however, these effects have barely been investigated in the hypercholesterolemic heart.

## 3. Functional Effects of Hypercholesterolemia on the Heart

Hypercholesterolemia has been shown to exert direct myocardial effects independent of the development of atherosclerosis in both clinical [40, 41] and preclinical studies [7, 8]. These effects include impaired cardiac performance and contractile dysfunction [6, 7], aggravated ischemia/reperfusion injury [9], and diminished adaptation to ischemic stress [10, 11, 42] (Figure 1).

### 3.1. Cardiac Contractile Dysfunction

Observations in human clinical trials and animal models suggest a direct effect of cholesterol on myocardial contractile function leading to impaired diastolic and in some cases also systolic function [6–8, 43–45]. Although the symptoms of hypercholesterolemia are not pronounced initially and the disorder may be dormant for a long time, preclinical diastolic dysfunction shows a clear progression to heart failure [46]. Development of heart failure aggravates with advancing age and contributes to age-related morbidity and mortality [47].

The direct effects of cholesterol exposure on cardiomyocyte function were demonstrated in a cell culture model; that is, elevation of membrane cholesterol content in ventricular cardiomyocytes resulted in decreased cytosolic calcium levels and impaired cardiac myocyte contractility [48]. This has been confirmed in cholesterol-fed rabbits and rats, showing apparent contractile dysfunction characterized by decreased maximum rate of shortening, decreased rate of relaxation, and increased left ventricular end-diastolic pressure [7, 8, 10, 44]. Moreover, impairment of cardiac performance assessed by measuring aortic flow was also demonstrated in hearts isolated from hypercholesterolemic apoB100 transgenic mice [6]. Hypercholesterolemia-induced cardiac dysfunction was further confirmed by echocardiography in humans [43, 45, 49].

It has been shown previously that myocardial oxidative/nitrative stress induced by hypercholesterolemia significantly contributes to the development of cardiac dysfunction [6, 8, 50]. However, the exact underlying molecular mechanisms are still not entirely clear. One possible mechanism is the oxidation of contractile proteins [8, 51, 52]. In failing human heart samples, for instance, there is an obvious oxidation and nitrosylation of tropomyosin and actin, showing a positive correlation with diminished contractile function indicated as a decrease in ejection fraction [51]. Moreover, increased protein oxidation was confirmed in the hearts of hypercholesterolemic rats [8].

### 3.2. Aggravated Ischemia/Reperfusion Injury and Attenuated Stress Adaptation

Hypercholesterolemia facilitates the risk of atherosclerosis and subsequent myocardial infarction. The current treatment of myocardial infarction includes the attempt to reopen the occluded coronary artery (termed reperfusion) in a timely manner by coronary intervention procedures or thrombolytic therapies in order to reduce infarct size, which is one of the major determinants of long term complications and survival. Thus the majority of patients with acute myocardial infarction undergo ischemia/reperfusion injury. However, the myocardium is remarkably good at adapting to ischemic conditions by triggering endogenous adaptive mechanisms against ischemia/reperfusion injury. One of the most powerful endogenous adaptive cardioprotective mechanisms is ischemic preconditioning, that is, when brief exposure to repetitive ischemia/reperfusion cycles markedly enhances the ability of the heart to withstand a subsequent, potentially lethal ischemic attack [53]. Another possible intervention is termed ischemic postconditioning when the initial phase of reperfusion is interrupted with short periods of ischemia [54]. In addition, remote conditioning has also been introduced when cardioprotection is achieved by exposing an organ at a distance from the heart to ischemia/reperfusion insults [55]. To date, a very large number of preclinical and clinical studies are available suggesting that hypercholesterolemia enhances the severity of ischemia/reperfusion injury and interferes with the endogenous cardioprotective mechanisms. The mechanisms of ischemia/reperfusion injury and endogenous cardioprotection as well as the effect of hypercholesterolemia on these phenomena have been extensively reviewed elsewhere [56–58].

## 4. Modulation of Hypercholesterolemia-Induced Oxidative/Nitrative Stress

Modulation of oxidative/nitrative stress in hypercholesterolemia can be approached by at least 3 different ways (Figure 1). First, cholesterol-lowering therapies should be effective in attenuation of oxidative/nitrative stress due to their trigger-eliminating effect. Second, the mechanism of action of several drugs used for cholesterol-lowering is complex and may involve antioxidant properties. The second approach may be the most obvious, that is, the application of antioxidant molecules and especially natural products to reduce oxidative/nitrative stress. Third, support or induction of endogenous enzymatic antioxidant systems or inhibition of the prooxidant enzymes may be a feasible way to control cardiac oxidative/nitrative stress in hypercholesterolemia. These distinct mechanisms of action are often combined in the case of certain modulators. In this section we discuss various pharmaceuticals, nutraceuticals, some novel approaches, and even physical exercise as potential modulators of hypercholesterolemia-induced oxidative/nitrative stress.

### 4.1. Pharmaceuticals

To the best of our knowledge, there are no drugs on the market approved to specifically target cardiac oxidative/nitrative stress induced by hypercholesterolemia. Nevertheless, cholesterol-lowering drugs are excessively prescribed for patients presenting hypercholesterolemia and due to their cholesterol-lowering effect they should have a secondary attenuating effect on hypercholesterolemia-induced oxidative/nitrative stress. Interestingly, several of these drugs have also been implicated in directly modulating oxidative/nitrative stress. Moreover, large clinical trials have shown that antihyperlipidemic agents, for example, statins [59], fibrates [60], and niacin [61], could reduce the incidence of cardiovascular events in hypercholesterolemic patients [59]. In addition, some drugs used for other indications than treatment of hypercholesterolemia (e.g., some antidiabetics and vasodilators) have also been demonstrated to attenuate hypercholesterolemia-induced oxidative/nitrative stress and deleterious cardiac consequences.

#### 4.1.1. Statins

Statins are a class of cholesterol-lowering drugs that inhibit the key enzyme, 3-hydroxy-3-methylglutaryl coenzyme A (HMG-CoA) reductase, of the endogenous cholesterol biosynthesis. Statins have therefore become established in the treatment of hypercholesterolemia and attained a central place in cardiovascular medicine [62]. However, there are potential side effects of statin therapy including skeletal muscle complaints and/or mild elevation of serum creatine kinase level and very rarely rhabdomyolysis [63]. The main members of statins on the market are lovastatin, rosuvastatin, simvastatin, atorvastatin, fluvastatin, pravastatin, and the newest addition, pitavastatin. By inhibition of the formation of mevalonate, the direct reaction product of HMG-CoA reductase and HMG-CoA, statins not only inhibit the formation of the end-product, cholesterol, but also they reduce the formation of other cholesterol pathway intermediers such as the 15-carbon isoprenoid farnesol or the 20-carbon isoprenoid geranylgeraniol which finally lead to reductions in protein prenylation, Coenzyme Q10 (see later), and dolichol synthesis. Besides lipid-lowering effects, statins have pleiotropic effects on different cell types. Some of these cholesterol-independent effects of statins involve improved endothelial function, stabilization of atherosclerotic plaques, and attenuation of oxidative stress and inflammation as well as inhibition of the thrombogenic response [64, 65]. Pleiotropic effects may play an important part in reducing cardiovascular mortality and morbidity and may act at multiple points in the complex cascade of events leading to atherosclerosis.

Regarding the antioxidative effect of statins, a recent study showed that, beside the cholesterol-lowering effect, simvastatin is able to ameliorate endothelial dysfunction through increasing NO bioavailability and through suppression of oxidative stress in a rat model of hypercholesterolemia [66]. Similar results were shown by Iliodromitis et al., [67] who found that a 3-week simvastatin treatment limits infarct size and attenuates oxidative and nitrosative stress both in normocholesterolemic and in hypercholesterolemic rabbits subjected to ischemia/reperfusion irrespective of the presence of postconditioning, while postconditioning was effective only in normocholesterolemic animals. According to another study [68], pravastatin, in contrast to same-dose simvastatin or postconditioning, was found to reduce infarction in hypercholesterolemic rabbits independently of lipid-lowering, potentially through eNOS activation and attenuation of oxidative/nitrative stress. The antioxidative effect of atorvastatin was also shown by several studies [69–72]. Moreover, in hyperlipidemic subjects with metabolic syndrome, atorvastatin is associated with a greater reduction in lipid markers of oxidation compared with pravastatin [73]. In contrast, Sodha et al. [74] found increased levels of myocardial biomarkers of oxidative stress in hypercholesterolemic swine treated with atorvastatin. Pitavastatin, the newest member of the HMG-CoA reductase inhibitor family, has shown improvements in both cardiovascular function and markers of oxidative stress [75], presumably via decreasing NADPH oxidase activation [64]. Fluvastatin was also shown to play a protective role against high-cholesterol-induced oxidative stress and DNA damage [76]. Taken together, the cardioprotective effect of almost every statin is associated with some pleiotropic effect which can decrease oxidative stress in the cardiovascular system.

#### 4.1.2. Ezetimibe

Ezetimibe, another lipid-lowering drug, is used in monotherapy or in combination with statins and is responsible for lowering intestinal cholesterol absorption by inhibiting the Niemann-Pick C1-Like 1 (NPC1L1) sterol transporter. Clinical studies showed that ezetimibe attenuates the markers of oxidative stress in hyperlipidemic subjects [77–80]. The drug is suggested to exert both cholesterol-dependent and independent actions [78–80]. Reduction of serum cholesterol results in decreased cholesterol influx to the cells thereby attenuating cholesterol-induced oxidative stress. For instance, ezetimibe reduced hepatic cholesterol level in obese male mice which in turn attenuated oxidative stress via downregulation of NADPH oxidases, cytochrome P4502E1, and beta oxidation [81]. In addition to its cholesterol-lowering action, ezetimibe may exert a direct cholesterol-independent effect on cells [78–80, 82]. A feasible explanation is that NPC1L1 is widely expressed in many other tissues including liver, kidney, muscle, and heart [83]. Cell culture studies support the possible direct effect of ezetimibe on cellular oxidative/nitrative stress, for example, knockdown of NPC1L1 in hepatocytes attenuated ROS production [84]. Based on these findings it is plausible to speculate that ezetimibe has a direct impact on the heart and may decrease cholesterol-induced oxidative stress. Nevertheless, direct cardioprotective effect of ezetimibe has not yet been investigated in the literature; thus preclinical studies needed to address this issue.

#### 4.1.3. Niacin

Although niacin (nicotinic acid or Vit B_3_) is a potent lipid-lowering agent when used in pharmacologic doses, its clinical use is limited by side effects, so it can be applied particularly in combination to treat marked dyslipidemia with strict monitoring ([85], for review see [86]). Niacin administration was shown to reduce markers of serum oxidative stress and increase antioxidant paraoxonase-1 in patients with hypercholesterolemia [87]. A randomized controlled clinical trial reported that niacin improves both 6- and 15-year mortality of patients after myocardial infarction with or without metabolic syndrome thereby suggesting a cardioprotective effect for niacin [88]. Direct cardioprotective effect of niacin was reported in preclinical studies as well [89–92]. However, evidence is not available to clarify the potential beneficial effect of niacin in cholesterol-induced cardiac stress.

#### 4.1.4. Fibrates

Fibrates (amphipathic carboxylic acid derivatives) lower plasma triglyceride and small LDL level and increase high density lipoprotein (HDL) concentration; thus fibrates are particularly used to treat combined dyslipidemia related to diabetes or metabolic syndrome [93]. This class of drugs acts on nuclear peroxisome proliferator-activated receptor *α* (PPAR*α*), leading to transcriptional changes [94]. Studies revealing the effect of fibrates on cholesterol-induced cardiac oxidative stress are not available in the literature. Nevertheless, some investigations have reported an antioxidant effect of fibrates. A subcohort clinical trial has demonstrated that 3-week fenofibrate treatment reduced ox-LDL and 8-isoprostane, systemic markers of oxidative stress in patients with hypertriglyceridemia [95]. In accordance with these results, fenofibrate decreased ox-LDL with a modest reduction in cholesterol level in healthy normolipidemic older adults [96]. Fibrates may exert antioxidant actions independently from their lipid-lowering effect. For instance, Sugga et al. have reported that* in vivo* administration of fenofibrate and clofibrate before* ex vivo* myocardial ischemia/reperfusion reduced infarct size and oxidative stress [97]. Others have shown antioxidant and cardioprotective effects of fibrates against development of ventricular hypertrophy associated with oxidative stress [98, 99]. Furthermore, fibrates prevent endothelial dysfunction by ameliorating oxidative stress, without affecting plasma lipid levels [100, 101]. It has been also published that knockout of PPAR*α*, the ligand of fibrates, leads to oxidative stress associated with cardiac dysfunction [102]. In contrast, fenofibrate failed to improve lipotoxic cardiomyopathy [103], and high dose fibrate induced cardiac damage in healthy rats [104]. Taken together, the studies focusing on antioxidant action of fibrates suggest a potential beneficial effect on cholesterol-induced cardiac oxidative stress, although this needs to be confirmed in further studies.

#### 4.1.5. Other Pharmaceuticals

There are some available drugs, which are basically used to treat other diseases than hypercholesterolemia and which have beneficial effects on high-cholesterol-induced cardiac consequences.

Rosiglitazone is an antidiabetic drug; however, it also improves blood cholesterol level in hypercholesterolemic models [105, 106]. It was reported that the drug prevents the development of high-cholesterol-induced cardiac hypertrophy [105] and ameliorates postischemic recovery and nitrative stress in the heart of high-cholesterol-fed rabbits [107]. The latter research group has also shown that rosiglitazone alleviates aggravated postischemic myocardial injury and myeloperoxidase upregulation caused by hypercholesterolemia, independently from the lipid-lowering action [108]. Rosiglitazone has a direct antioxidant effect on cardiomyocytes [109] and has been demonstrated to prevent the upregulation of cardiac NOX4 in a high-fat high-sugar diet, streptozotocin-induced diabetic model associated with dyslipidemia [110]. These findings make rosiglitazone a promising modulator of cholesterol-induced cardiac oxidative stress.

Increasing the level of glucagon-like peptide-1 by administration of analogues or by inhibiting dipeptidyl peptidases is used for the treatment of diabetes and has recently emerged as a potential cardioprotective approach [111]. Specifically, a glucagon-like peptide-1 agonist was shown to reverse cardiac dysfunction induced by high-fat diet [112] and a dipeptidyl peptidase inhibitor was found to improve cardiac dysfunction and oxidative stress in high-fat high-fructose-fed mice [113].

The vasodilator fasudil (a potent rho kinase inhibitor) was shown to lower serum cholesterol level and decrease cardiac oxidative stress by enhancing antioxidants in hypercholesterolemic rat hearts [114]. The group has reported that fasudil restores cardiac eNOS and NO level diminished by high-cholesterol diet [114]. Interestingly, even in the state of hypercholesterolemia, fasudil can induce both preconditioning and postconditioning [115]. Moreover, fasudil restores the cardioprotective effect of ischemic postconditioning in rats with hypercholesterolemia [116].

### 4.2. Nutraceuticals

In spite of the extensive research in cardiology, particularly in ischemic heart diseases, only very few new cardioprotective drugs have found their way into clinical practice [117]. Therefore, there is a growing interest in nutraceuticals to treat and also prevent certain cardiac diseases or restore the injured adaptation of the heart. Nutraceuticals is a term that refers to a wide range of products including but not limited to natural herbal products, dietary supplements, isolated nutrients, and special diets. Since there is also a growing desire among potential customers to consume natural dietary products instead of chemically synthesized compounds, the importance of these nutraceuticals is being further emphasized.

Natural food substances have the potential to alter biological functions of the cells by mechanisms enhancing the endogenous antioxidant systems or through altering the redox signaling status of the cell. This is often related to the unique composition of different antioxidant compounds in the various nutraceuticals. These products could be beneficial in pathological conditions where oxidative stress plays an important role.

#### 4.2.1. Antioxidant Vitamins

A number of preclinical studies have demonstrated that classical antioxidant vitamins including beta carotene (Vit A precursor), folic acid (Vit B_9_) vitamin C, and vitamin E administered alone or in combination with other drugs or in multivitamin preparations could improve serum lipid profile and myocardial oxidative/nitrative stress in hypercholesterolemia [18, 118–126]. Moreover, low dose beta carotene has been shown to improve cardiac function and reduce myocardial oxidative stress after ischemia/reperfusion injury in hearts of rats [63]. In contrast, vitamin C alone or combined with vitamin E failed to reduce myocardial infarct size in an open chest rabbit model [127]. Indeed, Trolox, a vitamin E analogue did not reduce infarct size but accelerated functional recovery after myocardial infarction in porcine hearts [128]. In addition, we have previously shown that a multivitamin preparation supplemented with phytosterols decreased serum cholesterol level in hypercholesterolemic rats; however, it did not reduce infarct size either in normocholesterolemic or in hypercholesterolemic hearts of rats [118]. In contrast, preconditioning with vitamin E has been shown to improve postischemic contractile and vascular functions in hearts of rats after ischemia/reperfusion injury [129]. To the best of our knowledge, the effects of antioxidant vitamins have not been tested on cardioprotection conferred by ischemic pre- or postconditioning. However, decrease of oxidative/nitrative stress by antioxidant vitamins could be a potential therapeutic target in the restoration of cardioprotective adaptive mechanisms lost in hypercholesterolemia.

Despite a number of preclinical studies proving beneficial effects of antioxidant vitamins on cardiac pathologies in hypercholesterolemia, clinical trials investigating the effects of antioxidant vitamins on cardiovascular morbidity and mortality have been disappointing. The complex reasons that might explain the translational failures of preclinical results into clinical therapy are discussed in detail in recent reviews [130–132] and include the following: (i) oxidative/nitrative stress is just a late consequence of cardiac pathologies and not the primary cause; (ii) pathogenesis of cardiovascular diseases is complex and increased oxidative/nitrative stress is not the only cause of these disorders; (iii) antioxidant vitamin therapy is not able to reduce oxidative/nitrative stress due to inappropriate study design (inappropriate patient selection, failure in the administration route, suboptimal time and duration of antioxidant therapy, poor target specificity, and potential interaction with other drugs, etc.); (iv) a single antioxidant therapy is not enough to overcome increased oxidative/nitrative stress; (v) antioxidant molecules might have harmful effects on physiological processes or compensatory mechanisms in pathologic conditions induced by oxidative/nitrative stress.

The lack of beneficial effects of antioxidant vitamins on cardiovascular pathologies in clinical trials does not disprove that oxidative/nitrative stress plays a crucial role in cardiac pathologies in hypercholesterolemia. These clinical trials challenge us to develop better antioxidant approaches and better preclinical models as well as to design more appropriate clinical trials considering the aforementioned reasons of translational failure.

#### 4.2.2. Coenzyme Q

Coenzyme Q10 (CoQ10), also called ubiquinone or ubiquinol, is an isoprene derivative and an alternative product of the mevalonate/cholesterol pathway. CoQ10 is an endogenous nonenzymatic lipophilic antioxidant and free radical scavenging molecule playing a role in the mitochondrial electron transport in the inner mitochondrial membrane [133]. Moreover, CoQ10 has been reported to play part in many levels of the redox control of cellular signaling; in fact the autoxidation of its semiquinone form, generated in various membranes during electron transport, can be a primary source for hydrogen peroxide production, which activates transcription factors [134]. Due to its localization in the mitochondria, it plays a key role in the cellular bioenergetics especially in tissues with high energy requirements such as the myocardium which is extremely sensitive to CoQ10 deficiency [133, 135]. CoQ10 has been shown to decrease the levels of proinflammatory cytokines and to reduce LDL oxidation and consequently the progression of atherosclerosis. It also decreased ischemia/reperfusion injury after myocardial infarction [135, 136]. Significant improvement has been demonstrated in clinical and hemodynamic parameters and in exercise tolerance in patients given adjunctive CoQ10 in various trials conducted in patients suffering from heart failure, hypertension, ischemic heart disease, and other cardiac illnesses [135–137]. Therefore, CoQ10 could be a potential therapeutic molecule in heart diseases.

Due to the common biosynthetic pathway of cholesterol and CoQ10, the HMG-CoA reductase inhibitor statins may potentially reduce the levels of CoQ10 in different tissues [138]. Indeed, it has been reported that CoQ10 levels in the plasma, platelets, and lymphocytes were decreased after statin treatment [138]. Some papers indicated that CoQ10 depletion during statin therapy might be associated with subclinical cardiomyopathy and this situation is reversed upon CoQ10 treatment [138]. In contrast, a preclinical study reported that coadministration of CoQ10 with simvastatin impaired mitophagy and cardioprotection after ischemia/reperfusion injury in mice and cardiomyocytes [139]. These results raise the concern that CoQ may interfere with the anti-ischemic benefit of statins mediated through stimulation of mitophagy. Therefore, patients treated with statins should be also monitored for their CoQ10 status and clinicians should be aware of the aforementioned interaction between statins and CoQ10 as well as the ability of statins to impair skeletal muscle and myocardial bioenergetics [138].

#### 4.2.3. Flavonoids

Flavonoids are a large family of natural polyphenolic compounds in the human diet and their beneficial effect on cardiovascular diseases is widely studied. These compounds favorably affect a wide range of biological processes. Beside their antioxidant capacity, flavonoids improve lipid profile and have anti-inflammatory, antiplatelet, and antithrombotic effects as well.

*Green Tea Catechins (GTC)*. Catechins are abundant polyphenols in green tea. GTC were shown to reduce blood cholesterol level in both animals [140–143] and humans [144–147]. In addition, GTC reduced accumulation of cholesterol in the rat myocardium in a state of diabetic dyslipidemia [141]. In a double-blind, placebo-controlled clinical study, obese, hypertensive patients had decreased LDL-cholesterol and increased HDL and total serum antioxidant levels after 3 months of GTC supplementation [148]. Mehra et al. demonstrated that catechin hydrate improved high sucrose, high-fat-induced cardiac lipid peroxidation and activity of antioxidant enzymes [149]. GTC were reported to beneficially affect high-fructose diet-associated hypercholesterolemia and cardiac signaling pathways related to lipid metabolism and inflammation [150]. Age-related cardiac oxidative stress was also improved by GTC [151]. The effect of GTC on hypercholesterolemia associated with cardiac dysfunction is not known, but it was reported that GTC ameliorates myocardial function after ischemia [152, 153] in pressure-induced chronic heart failure [154] and in autoimmune myocarditis as well [155].

*Troxerutin*. Troxerutin can be isolated from the Japanese pagoda tree (*Sophora japonica*). To the best of our knowledge, the antioxidant effect of troxerutin on the heart was first described in an ischemia/reperfusion model [156]. Geetha et al. have investigated the cardiac impact of troxerutin in high-fat high-fructose mice model. They have found that troxerutin reduces cholesterol content and oxidative stress markers in both the plasma and the heart and increases cardiac enzymatic and nonenzymatic antioxidant levels [157]. Moreover, the same group have shown in the same dyslipidemic model that troxerutin reverses fibrotic changes in the heart and improves cardiac function probably by reducing ROS production [158].

*Quercetin*. The cardioprotective effect of the dietary flavonoid quercetin (found in many fruits, vegetables, leaves, and grains) against ischemia/reperfusion injury is well studied [159–163]; however, few studies are available in the literature regarding the impact on hypercholesterolemia-induced oxidative stress in the heart. It was reported that quercetin ameliorates left ventricular function, collagen deposition, and inflammatory cell infiltration in rats with metabolic syndrome [164]. In that study, quercetin increased the expression of the transcription factor Nrf2 and the enzyme heme oxygenase as protective proteins against oxidative stress. Interestingly, quercetin exerted a cardioprotective effect without reducing plasma cholesterol level thereby suggesting a direct cardiac effect. In other studies, quercetin has been shown to reduce serum LDL and increase HDL cholesterol level in hypercholesterolemic rats [165] and lower total cholesterol in rabbits fed a high-cholesterol [166] and high-fat [167] diet. Ulasova et al. showed that quercetin improves lipid profile of ApoE knockout mice and prevents ventricular hypertrophy without affecting myocardial function [168]. In a cadmium-induced toxicity study, quercetin was found to reduce cardiotoxicity and dyslipidemia by attenuating cardiac oxidative stress and lipid parameters [169].

*Rutin*. Rutin (the glycoside between the quercetin and the disaccharide rutinose) improves cholesterol level in different hypercholesterolemic [170, 171] and dyslipidemic [172, 173] rodent models. Panchal et al. have shown that rutin supplementation ameliorates blood cholesterol level, cardiac structure, function, inflammation, and oxidative stress related to high carbohydrate high-fat-induced dyslipidemia [173]. In a streptozotocin-induced diabetes model associated with hyperlipidemia, rutin attenuated serum cholesterol level and myocardial necrosis and improved left ventricular dysfunction [174]. It was also reported that rutin exerts cardioprotection by attenuating oxidative stress and dyslipidemia induced by high fluoride administration in rats [172].

*Silymarin*. Silymarin is a mixture of three flavonolignans (silibinin, silydianin, and silychristin) extracted from milk thistle seeds (*Silybum marianum*). Krečman et al. demonstrated that silymarin prevented the development of hypercholesterolemia in high-cholesterol-fed rats [175]. They have also shown that silibinin itself was not as effective as silymarin. Others also reported that silymarin or its fraction decreased total and LDL cholesterol and attenuated oxidative stress in the plasma [176–178]. The effect of silymarin on hypercholesterolemia-induced myocardial oxidative stress is not yet investigated, but some studies indicate that silymarin exerts cardioprotection against high-cholesterol-mediated oxidative stress. Silymarin attenuates myocardial ischemia/reperfusion injury by modulating oxidative stress [179] and silibinin has a direct antioxidant effect on H9c2 cardiac cells against oxidative stress [180]. Moreover, silibinin was reported to improve both plasma and cardiac cholesterol content and attenuate oxidative markers and degenerative changes in the heart of animals exposed to arsenic [181]. In this study, silibinin prevented cardiac oxidative stress by inhibiting the induction of prooxidants (e.g., NOX2 and NOX4) and enhancing antioxidants.

*Naringin and Hesperidin*. Naringin and hesperidin are natural flavonglycosides in citrus fruits. Many studies reported hypocholesterolemic effect of naringin and hesperidin in animal models [182–188] and in a clinical study [189]. However, in moderately hypercholesterolemic subjects, naringin and hesperidin failed to lower serum cholesterol [190]. Alam et al. have published that naringin improves ventricular diastolic dysfunction, cardiac inflammatory cell infiltration, and plasma cholesterol in high carbohydrate, high-fat-fed rats [182]. Recently, naringin has been shown to protect against hypercholesterolemia-induced oxidative stress in the heart [191]. The study has demonstrated that naringin ameliorates cardiac lipid accumulation and cardiac oxidative stress markers by enhancing enzymatic and nonenzymatic antioxidants. Moreover, tissue and serum markers of cholesterol-induced cardiac damage were also attenuated by naringin supplementation [191]. Although hesperidin was reported to protect against cardiac injury induced by doxorubicin [192] or ischemia [193, 194], its role in cholesterol-induced cardiac oxidative stress is not known.

#### 4.2.4. Resveratrol

Pleiotropic beneficial effects of the polyphenol resveratrol (food sources include the skin of grapes, blueberries, raspberries, and mulberries) have been extensively studied and many publications indicate its protective function on cholesterol-induced cardiac oxidative stress. A direct antioxidant effect of resveratrol was demonstrated on H9c2 cardiac cells [195]. Louis et al. have demonstrated that resveratrol ameliorates cardiac relaxation dysfunction in high-fat-fed rats with hypercholesterolemia [196]. They have also shown reduced oxidative stress and inflammatory markers in the serum as a result of resveratrol treatment [196]. In hypercholesterolemic rats with normal cardiac function, resveratrol improved postischemic recovery of the heart [197]. Large animal studies from the same research group reported that resveratrol improved high-cholesterol and chronic ischemia-induced cardiac dysfunction and oxidative damage of myocardial proteins [198–200]. The group also described that resveratrol improved high-cholesterol-induced myocardial dysfunction without decreasing protein oxidation in the absence of ischemia [198]. Chu et al. have shown that red wine ameliorates cardiac dysfunction and oxidative stress in hypercholesterolemic swine subjected to chronic ischemia [50]. Resveratrol was reported to improve lipid profile and cardiac dysfunction related to hyperlipidemia in streptozotocin-induced diabetes [201]. Finally, these results may promote new studies focusing on cholesterol-induced cardiac oxidative stress.

#### 4.2.5. Grape Seed

Grape seed and skin are rich in natural antioxidants, so these are possible supplements to alleviate hypercholesterolemia-induced oxidative stress. In a high-fat-induced obesity model, grape seed extract was shown to improve lipid profile and prevent postischemic heart dysfunction and cardiac lipid accumulation along with attenuated oxidative stress [202, 203]. Lee et al. reported that grape skin ameliorates total serum antioxidant capacity of rats with high-fat diet and low-fat diet [204]. Antioxidant effect of grape seed was tested on cardiomyoblast H9c2 cell culture [205], where it increased endogenous antioxidant systems and prevented ROS-induced apoptosis [205].* In vivo* pretreatment with grape seed proanthocyanidins improved postischemic functional recovery and reduced ROS production in normocholesterolemic rats [206] and partially restored the harmful cardiac effects of hypercholesterolemia via their ability to reduce ROS in the myocardium [207].

#### 4.2.6. Sour Cherry Seed Extract

Based on the observation that cherries contain bioactive phytochemicals, for example, phenolics and anthocyanins, which are reported to possess antioxidant, anti-inflammatory, anticancer, antidiabetic, and antiobesity properties, Tosaki and his group hypothesized that the seed kernel of sour cherry (*Prunus cerasus*) may contain different bioactive constituents [208]. They demonstrated that kernel extract obtained from sour cherry seed improves postischemic cardiac functional recovery and the incidence of ventricular fibrillation and tachycardia in isolated working rat hearts [209]. Moreover, sour cherry seed extract-induced improvement in cardiac function after ischemia/reperfusion along with decreased atherosclerotic plaque formation and infarct size was also observed in hypercholesterolemic New Zealand rabbits fed a 2% cholesterol-enriched diet for 16 weeks [210]. In this model, the authors demonstrated an increased HO-1 and cytochrome c oxidase III protein expression following administration of sour cherry seed extract as a possible mechanism of action [210, 211].

#### 4.2.7. Spices

Aqueous extracts of certain spices including garlic (*Allium sativum*), ginger (*Zingiber officinale*), and cayenne pepper (*Capsicum frutescens*) as well as their mixture were shown to attenuate cardiac lipid peroxidation induced by high-cholesterol, high-fat diet in a rat model of hypercholesterolemia [20]. Moreover, in the same study, hypercholesterolemia-induced decrease in the myocardial activities of antioxidant enzymes (i.e., SOD, glutathione peroxidase, and glutathione reductase) was also markedly attenuated by administration of the individual spices as well as their combination [20].

#### 4.2.8. Red Palm Oil

Red palm oil (RPO) is a product from the fruits of the oil palm tree (*Elaeis guineensis*). RPO depending on the producer consists of about 51% saturated fatty acids (SFAs), 38% monounsaturated fatty acids (MUFAs), 11% polyunsaturated fatty acids (PUFAs), and a spectrum of antioxidative carotenoids with tocopherols and tocotrienols as the major constituents [212]. Other minor components present in this oil are ubiquinones (mainly CoQ10) and phytosterols. Red palm oil is therefore a natural carotenoid rich oil that has the potential to act as a very potent antioxidant [213]. Red palm oil contains the highest concentration of tocotrienols compared with other vegetables or plants and Serbinova et al. showed that tocotrienols can be 40–60 times more potent as antioxidants than tocopherols [214].

Van Rooyen's research group investigated the effect of dietary administration of RPO on the heart. They showed that prolonged dietary feeding with RPO-supplemented diet (~7 g RPO/kg diet) protected the heart against ischemia/reperfusion injury in rats. They used isolated heart models, perfused both in Langendorff technique [215–218] and working mode [212, 219], showing the vascular-independent direct cardioprotective effects of RPO. RPO administration improved cardiac function during reperfusion [212] and decreased infarct size [215, 216]. The real advantage of RPO administration became apparent when RPO was given to hyperlipidemic rats. Hyperlipidemia was induced by feeding animals with 2% cholesterol-enriched diet for 5–9 weeks. This model was characterized by mild cholesterol elevation but a marked decrease of cardiac performance. RPO was able to markedly increase aortic output recovery after 25 min global ischemia [219, 220], or infarct size [215] after 30 min global ischemia, showing the protective effect of RPO in the presence of comorbidities.

The proposed mechanism by which RPO exerts its cardioprotective effect in animals fed high-cholesterol diet is not fully understood. Supplementation with RPO in the presence of potentially harmful cholesterol showed no significant difference in serum cholesterol level; therefore, its protection cannot be explained by the cholesterol-lowering effect of RPO. It is proposed that the protective effect of RPO in high-cholesterol diet may be associated with either the RPO antioxidant characteristics and/or changes in the fatty acid composition of the myocardium during ischemia/reperfusion.

### 4.3. Promising Novel Approaches to Modulate Hypercholesterolemia-Induced Oxidative Stress

#### 4.3.1. miR Modulation

Only 3% of the human genome codes for proteins and the remaining part consist of noncoding RNAs including short microRNAs (miRNAs, miRs; approximately 18–25 nucleotides in length) [221, 222]. miRNAs can inhibit the translation or promote mRNA degradation by binding to specific mRNAs according to the complementarity of their seed sequences [223]. Individual miRNAs may simultaneously target multiple mRNAs. However, the expression of individual mRNAs can be regulated by multiple miRNAs. Therefore, miRNAs may act as fine tuners or as on/off switchers of gene expression [223, 224]. Dysregulation of miRNAs in pathological conditions may alter gene networks; therefore miRNA replacement or antisense inhibition therapy offers a new approach to treating diseases by modulating gene pathways rather than single molecular targets [222].

In recent years, a growing body of evidence has demonstrated that miRNAs play a role in the development of numerous cardiovascular diseases. Several excellent reviews focus on miRNAs as diagnostic markers and potential therapeutic targets in cardiac pathologies including acute coronary syndrome [221, 225] and remodelling after myocardial infarction as well as heart failure [25, 221, 224, 226–228] and their risk factors including hypercholesterolemia [229], diabetes mellitus [230, 231], arterial hypertension [232, 233], atherosclerosis [234, 235], and aging [227].

Hypercholesterolemia is a well-known risk factor of cardiovascular diseases and it leads to increased oxidative/nitrative stress in the myocardium. Experimental data are very limited on the regulatory role of miRNAs in hypercholesterolemia-induced oxidative/nitrative stress in cardiac pathologies. We have previously shown that the myocardial downregulation of miR-25 results in the upregulation of NADPH oxidase 4 (NOX4) mediating oxidative/nitrative stress and subsequent myocardial dysfunction in male hypercholesterolemic rats [8] (Table 1). Moreover, in a recent study, decreased circulating miRNA-25 level has been related to the level of oxidative stress indicators in septic patients and the clinical accuracy of miRNA-25 for sepsis diagnosis has been reported to be better than C-reactive protein [236]. In addition, downregulation of miR-25 expression has been demonstrated in cardiac hypertrophy induced by transverse aortic constriction surgery in mice; however, there is no data published on increased oxidative/nitrative stress or cholesterol levels in that study [237]. Furthermore,* in vivo* inhibition of miR-25 by a specific antagomir resulted in the spontaneous development of cardiac dysfunction and sensitized the myocardium to develop heart failure in a Hand2-dependent manner [237]. In contrast, inhibition of overexpressed miR-25 was reported to ameliorate contractile dysfunction by improving sarco/endoplasmic reticulum Ca^2+^-ATPase (SERCA)2a activity and Ca^2+^ handling in chronic heart failure induced by transverse aortic constriction surgery in mice as well as in failing human heart samples; however, data on the presence of hypercholesterolemia in humans were lacking in this study [238].

It is well known that hyperlipidemia, obesity, metabolic syndrome, and heart failure are associated with abnormal cardiac metabolism. In obese mice, a heart specific miRNA, miR-208a, has been reported to negatively regulate mediator complex subunit 13 (MED13), which controls transcription by thyroid hormone and other nuclear hormone receptors [30] (Table 1). Indeed, cardiac-specific overexpression of MED13 or pharmacologic inhibition of miR-208a in mice has been demonstrated to confer resistance to high-fat diet-induced obesity and improve systemic glucose tolerance [30] (Table 1). Moreover, mice genetically lacking miR-378 and miR-378^*∗*^ have been shown to be resistant to high-fat diet-induced obesity and exhibit enhanced mitochondrial fatty acid metabolism and elevated oxidative capacity of insulin-target tissues [31] (Table 1). Interestingly, MED13 and carnitine O-acetyltransferase, a mitochondrial enzyme involved in fatty acid metabolism, are among the many targets of miR-378 and miR-378^*∗*^ [31] (Table 1). Thus, these miRNAs provide potential therapeutic targets in hyperlipidemia and metabolic disorders, although their myocardial function needs to be further investigated.

High level of LDL cholesterol and low level of HDL cholesterol are both well known as independent risk factors of coronary artery diseases. miRNAs have been shown to regulate lipoprotein metabolism and their pro-/antiatherogenic effects at many levels in different tissues as reviewed by others [25, 222, 229]. Cholesterol efflux from cells is the first step in reverse cholesterol transport to the liver carried out by HDL. miR-33 has been reported to modulate cholesterol efflux by repressing the expression of ATP-binding cassette transporter (ABC) A1 in the liver and ABCA1 as well as ABCG1 in peripheral tissues [25] (Table 1). Moreover, anti-miR-33 therapy was shown to induce the expression of ABCA1 in macrophages in atherosclerotic plaques thereby reducing the plaque size and local inflammation in mice [26] (Table 1). In addition, anti-miR-33 therapy in nonhuman primates increased the expression of miR-33 target genes involved in fatty acid oxidation and reduced the expression of genes associated with fatty acid synthesis in the liver resulting in a marked suppression of plasma VLDL triglyceride levels [27] (Table 1). Another miRNA, miR-144, has been reported to decrease hepatic ABCA1 expression and plasma HDL cholesterol levels [28] (Table 1). Moreover, miR-223 has been demonstrated to reduce HDL cholesterol uptake by decreasing the expression of scavenger receptor B1 and to decrease cholesterol biosynthesis through the direct repression of HMG-CoA synthase 1 in the liver of ApoE^−/−^ mice [29] (Table 1). Furthermore, genetic ablation of miR-223 resulted in elevated hepatic and plasma total cholesterol levels as well as increased HDL cholesterol levels and particle size [29] (Table 1). Interestingly, aortae of mice fed with high-fat diet for 6 weeks and human hypercholesterolemic sera showed decreased let-7g expression [24] (Table 1). In the same study a negative feedback regulation has been identified between oxidized LDL receptor 1 and let-7g in primary human aortic smooth muscle cells [24] (Table 1).

Certain microRNAs have been implicated in cellular responses to oxidative/nitrative stress in cardiovascular pathologies in preclinical studies [33, 238–241]. Many miRNAs, including miR-21 and miR-199a, have been reported to play a role in cardiomyocyte survival during ischemia [33, 242] (Table 1). Moreover, injection of AAV9 vectors expressing miR-199 and 590 into the peri-infarcted area of the myocardium could reduce infarct size and improve regeneration after myocardial infarction in mice [243]. We and others have shown that several miRNAs including miR-1, miR-21, miR-125b^*∗*^, miR-139-3p, miR-139-5p, miR-181a, miR-188, miR-192, miR-212, miR-320, miR-487b, and miR-532 play a role in the mechanism of ischemic preconditioning conferring cardioprotection after ischemia/reperfusion injury [32, 34] (Table 2). These miRNAs also drive the synthesis of important cardioprotective proteins including heat shock protein- (HSP-) 70, endothelial and inducible NOS, HSP-20, NAD-dependent deacetylase sirtuin-1 (Sirt1), and hypoxia-inducible factor 1a [34]. miRNAs are also associated with the protective effect of ischemic postconditioning against myocardial ischemia/reperfusion injury. Heart specific miR-1 and miR-133a have been associated with playing a role in the cardioprotection conferred by ischemic postconditioning through the regulation of apoptosis-related genes [35, 37]; however, the regulation of miR-1 by ischemic postconditioning is controversial [32, 35, 37] (Table 2). Another miRNA, miR-21, has been demonstrated to be implicated in ischemic postconditioning, though its role in cardioprotection is controversial [35, 36] (Table 2). In addition, loss of the miR-144/451 cluster function has been shown to limit the cardioprotective effect of ischemic preconditioning by upregulating Rac-1-mediated oxidative stress signaling [38]. Therefore, further preclinical and clinical studies are needed to investigate the role of miRNAs in ischemia/reperfusion injury and myocardial stress adaptation in healthy and diseased conditions, including hypercholesterolemia.

miRNAs exert control over diverse metabolic pathways and are frequently dysregulated in cardiovascular diseases. Thus, miRNAs have become a class of promising therapeutic targets. Until miRNA-based therapeutic interventions become a reality in clinical medicine many questions should be answered in preclinical and clinical studies [244]. Better technologies and more applicable* in vitro* and* in vivo* models of human diseases should be developed to identify and validate direct mRNA targets of miRNAs [244]. Furthermore, improved understanding of the mechanism of action in each tissue type is necessary. Moreover, development of organ specific delivery methods for miRNA mimics and anti-miR oligonucleotides are needed [244]. Nevertheless, assessment of the efficacy and safety including the analysis of the off-target effects of miRNA-based therapeutic tools and understanding of the long-term effects of miRNA modulation* in vivo* are of key importance in the future [244].

#### 4.3.2. Peroxynitrite Scavenging

Peroxynitrite is formed by the rapid reaction of superoxide and NO and is responsible for a variety of deleterious effects in cardiovascular pathologies [245]. Therefore, development of peroxynitrite scavengers or compounds catalyzing the decomposition of peroxynitrite to nontoxic products has been an emerging field in the last decade [245]. Formation of peroxynitrite in the heart of hypercholesterolemic animals has been demonstrated in various experimental models [6, 8, 246]. The beneficial effect of decomposition of cardiac peroxynitrite leading to improved cardiac function in experimental hypercholesterolemia was also shown [6, 246]. In isolated working hearts from Wistar rats or apoB100 transgenic mice fed with cholesterol-enriched diet, deterioration of cardiac function characterized by increased left ventricular end-diastolic pressure (LVEDP) or decreased aortic flow was demonstrated, respectively [6, 246]. Pretreatment of the animals with the peroxynitrite decomposition catalyst FeTPPS (5,10,15,20-tetrakis(4-sulfonatophenyl)porphyrinato iron (III), chloride) before isolation of the hearts resulted in an improved LVEDP and aortic flow, respectively [6, 246]. Whether application of peroxynitrite scavengers would reverse impaired conditioning in hypercholesterolemic animals remains to be addressed in future studies.

#### 4.3.3. Hydrogen

Recent advances in basic and clinical research have indicated that hydrogen gas is an important physiological regulatory factor with antioxidant, anti-inflammatory, and antiapoptotic protective effects, and thus the application of molecular hydrogen as a therapeutic medical gas in diverse disease conditions has become a feasible therapeutic strategy [247]. Hydrogen is suggested to be an efficient, nontoxic, highly bioavailable, and low-cost antioxidant supplement for patients with pathological conditions involving ROS-induced oxidative stress [248]. Therapeutic hydrogen can be applied by different delivery methods including inhalation of hydrogen gas, drinking hydrogen dissolved in water, and injection with hydrogen-saturated saline [247]. In the heart, hydrogen attenuated doxorubicin-induced heart failure in rats [249], cardiac dysfunction in streptozotocin-induced diabetic mice [250], rat cardiac cold ischemia/reperfusion injury [251], and left ventricular hypertrophy in spontaneous hypertensive rats [252] and exerted cardioprotective effects on isoproterenol-induced myocardial infarction in rats [253]. Moreover, inhalation of hydrogen attenuated increased serum cholesterol, cardiac superoxide production, and left ventricular remodeling induced by intermittent hypoxia in mice [254]. Similarly, consumption of hydrogen-rich water beneficially affected serum cholesterol status and oxidative stress markers in patients with potential metabolic syndrome or isolated hypercholesterolemia [255, 256]. These promising results should be confirmed.

#### 4.3.4. Miscellaneous Examples

Diphenyl diselenide has been recently reported to attenuate hypercholesterolemia-associated cardiac oxidative stress and increase antioxidants without affecting plasma level of cholesterol in LDL receptor knockout mice [257].

Local infiltration of neuropeptide Y to hypercholesterolemic swine heart subjected to ischemia was shown to ameliorate cardiac diastolic dysfunction probably by decreasing oxidative stress and fibrosis and increasing cell survival in the heart [258, 259].

### 4.4. Physical Activity

A dramatic decrease in an individual's physical activity is the most obvious change accompanied by western-type lifestyle and technical development in the industrial countries. Physical inactivity is a risk factor and promotes development of civilization diseases. It is widely accepted that physical activity positively influences a variety of clinical diseases including obesity, metabolic syndrome, dys- and hyperlipidemias, diabetes, and cardiovascular diseases [260, 261].

Hypercholesterolemia is accepted as one of the most important risk factors in the development of different vascular and heart diseases. Physical activity and change in lifestyle are the first choices in normalization of patient's high blood cholesterol level. The mechanisms by which physical activity prevents development of metabolic diseases are rather diverse. The primary effect of physical activity can be seen in a metabolic level. Due to higher energy demand, physical activity intensively increases weight loss. A weight loss of 10 percent can significantly lower the risk of cardiovascular diseases by reversing hyperlipidemia. Moreover, by increasing the catabolic rate of the body, physical exercise positively influences carbohydrate and lipid metabolism and blood lipid profile. Thus, exercise training is associated with increased reliance on lipids as an energy substrate, has a systemic lipid-lowering effect, and results in remodeling of skeletal muscle lipid metabolism toward increased oxidation and neutral lipid storage and turnover [262]. Physical activity has substantial effects on the liver metabolism as well, modifying lipoprotein levels to a more healthy composition. These effects can be enhanced by using concurrent dietary restrictions; for example, a low-cholesterol diet definitely helps to regulate lipid profile in the body. Regular participation in physical activity as well as a single exercise session can positively alter cholesterol metabolism [263]. Exercise is involved in increasing the production and action of several enzymes that function to enhance the reverse cholesterol transport system [263].

Nevertheless, physical activity has secondary effects on the prevention of development of metabolic diseases by, for example, modifying oxidative stress. Physical activity is believed to be a protective modulator of oxidative stress in hyperlipidemia; however, high intensity physical exercise itself can definitely increase oxidative stress in patients [264–266]. Two key free radicals are the most important during physical activity, that is, superoxide and NO. The exact sources of these radicals are not fully known; however, mitochondria are often cited as the predominant source of ROS in muscle cells. Investigators have often assumed that the increased ROS generation that occurs in muscle fibers during contractile activity is directly related to the elevated oxygen consumption. However, growing evidence argues against mitochondria being the dominant source of ROS production in skeletal muscle during exercise [264]. Another possible source is NADPH oxidase, which is normally quiescent, but when it becomes activated, during muscle contraction or when recruited for antimicrobial and proinflammatory events, it can generate large amounts of superoxide [264, 267, 268]. Since the discovery that contracting skeletal muscles produce ROS, many investigators have assumed that skeletal muscle provides the major source of free radical and ROS generation during exercise. Nonetheless, other tissues such as the heart, lungs, or blood may also contribute to the total body generation of ROS during exercise [264]. Thus the increased catabolic process together with multiplied oxygen consumption in both skeletal and cardiac muscles during exercise exacerbates superoxide production.

The other major free radical which contributes to oxidative/nitrative stress is NO. NO is responsible for the relaxation of vessels [269] and plays an important role in matching tissue perfusion to demand [270]. The release of nitric oxide by the endothelial cell can be upregulated by exercise [261, 271]. However, hypercholesterolemia impairs endothelial function (e.g., the NO-cyclic GMP-phosphodiesterase 5 pathway), limits shear stress-induced vasodilation, and is therefore expected to reduce exercise-induced vasodilation [272].

In addition to the modulation of ROS and RNS production, physical exercise may also affect the antioxidant defense systems. McCommis et al. have demonstrated that familial hypercholesterolemia reduces mitochondrial antioxidants, increases mitochondrial oxidative stress, and enhances the mitochondrial permeability transition response in the porcine myocardium [19]. They also showed that exercise training can reverse these detrimental alterations without altering serum cholesterol level [19].

In spite of the extensive research, the cardioprotective effect of exercise which is mediated by redox changes is still a question of debate. Several studies showed that hyperlipidemia impairs exercise capacity itself [273] or the effect of physical activity [272, 274, 275]. However, a number of exercise programs have effectively reversed hypercholesterolemia-induced changes mainly within the vasculature by improving NO bioavailability in both animal studies and humans [276–281].

The exact explanation why physical activity, which similarly to hyperlipidemia leads to an increased oxidative stress, is able to protect the heart in hypercholesterolemia is rather difficult. One possible answer is that during exercise an intensive but only a temporary increase of oxidative stress occurs resulting in a possibility for cardiac adaptation by an improved enzymatic and nonenzymatic antioxidant capacity, cytoprotection, aerobic capacity, training-induced muscular adaptation, mitochondrial biogenesis, and so forth [268]. In hypercholesterolemia with no exercise, the continuously elevated oxidative/nitrative stress, however, does not allow the completion of cardioprotective mechanisms. End-effectors of cardioprotection involve the activation of ATP-sensitive K^+^-channels (KATP). We have recently demonstrated that a cholesterol-enriched diet inhibited cardioprotection induced by KATP activators and that cholesterol diet may impair cardiac KATP channels [282]. It is well accepted that the opening of KATP channels generates ROS; however, an ambient oxidative state also modifies redox-sensitive KATP channels, as superoxide, hydrogen peroxide, and peroxynitrite open KATP channels in the heart. These results show that increased oxidative stress interferes with KATP channel function and therefore might explain why cardioprotection is lost in hyperlipidemia.

Increased physical activity can be also mimicked in experimental animal models by applying ventricular overdrive pacing of the heart, a method that was reported to induce both preconditioning and postconditioning by increasing the oxygen demand (relative hypoxia) instead of limiting oxygen supply (absolute hypoxia) in isolated heart models [283–285]. Peroxynitrite plays an important role in different conditionings induced by either ischemia or ventricular pacing [11, 286]. It is known that experimental hypercholesterolemia blocks the cardioprotective effect of postconditioning at least in part via deterioration of postconditioning-induced early increase in peroxynitrite formation during reperfusion [11]. Thus one can speculate that regular physical exercise is able to restore the protective effects of pre- or postconditioning in hypercholesterolemia via modifying cardiac oxidative stress and by beneficially altering lipid profile in the blood.

In addition to emphasizing the importance of regular exercise, novel future directions have been implicated to take advantage of exercise-induced benefits. Thus development of new “exercise mimetics” has a promising role in the future for treatment of patients [287].

## 5. Conclusions

Oxidative and nitrative stress has been implicated as a pathophysiological mechanism of cardiovascular diseases; however, there is still no breakthrough regarding the use of general antioxidant therapies in clinical practice. The possible reasons for these disappointing results and some promising aspects of potential antioxidant therapy have been discussed in detail recently [288]. Although hypercholesterolemia occurs frequently in the adult population, the number of publications investigating myocardial oxidative/nitrative stress and its cardiac consequences is relatively limited especially in humans. This is unfortunate, as patients with hypercholesterolemia are at an increased risk for severe pathological conditions such as myocardial infarction and heart failure and hypercholesterolemia has been shown to interfere with endogenous cardioprotective mechanisms. Therefore, finding proper approaches to beneficially affect hypercholesterolemia and its myocardial consequences is crucial. These reasons warrant further preclinical and clinical studies to better understand the pathological events in the heart relating to hypercholesterolemic conditions and to find the best approaches to interact. Moreover, many of the potential modulators of oxidative/nitrative stress require further development to optimize their effects for application in hypercholesterolemia.

## Figures and Tables

**Table 1:** Regulation of miRNAs in hyperlipidemia.

miRNA	Regulation of miRNA	Organ	Target	Regulation of target	Role	References
let-7g	down	Aorta	ox-LDL receptor 1	up	ox-LDL cholesterol uptake	[24]
miR-25	down	Heart	NOX4	up	Oxidative stress	[8]
miR-33	up	Liver, macrophages	ABCA1	down	Reverse cholesterol transport	[25]
miR-33	up	Peripheral tissues	ABCG1	down	Reverse cholesterol transport	[25, 26]
miR-33	down	Liver	Fatty acid synthesis	down	VLDL synthesis	[27]
miR-33	down	Liver	Fatty acid oxidation	up	VLDL synthesis	[27]
miR-144	up	Liver	ABCA1	down	Reverse cholesterol transport	[28]
miR-223	up	Liver	HMG-CoA synthase 1	down	Cholesterol biosynthesis	[29]
miR-223	up	Liver	Scavenger receptor B1	down	HDL cholesterol uptake	[29]
miR-208a	up	Heart	MED13	down	Glucose tolerance	[30]
miR-378 and miR-378^*∗*^	up	Liver	MED13	down	Glucose tolerance	[31]
miR-378 and miR-378^*∗*^	up	Insulin dependent tissues	Fatty acid oxidation	down	Obesity	[31]

ABC: ATP-binding cassette transporter; HDL: high density lipoprotein; VLDL: very low density lipoprotein; MED: mediator complex subunit; NOX: NADPH oxidase.

**Table 2:** miRNAs affected by ischemic pre- or postconditioning in the heart.

miRNA	I/R versus control	Ipre versus I/R	Ipost versus I/R
let-7b	down [32]	n.a. [32]	up [32]
miR-21	down [33]	up [34, 35]	n.a. [35], up [36]
miR-125b^*∗*^	down [32]	up [32]	up [32]
miR-139-3p	down [32]	up [32]	up [32]
miR-181a	down [32]	down [32]	up [32]
miR-199a	down [33]	up [34]	no data
miR-328	down [32]	n.a. [32]	up [32]
miR-335	down [32]	n.a. [32]	up [32]
miR-503	down [32]	n.a. [32]	up [32]
let-7e	n.a. [32]	n.a. [32]	up [32]
let-7i	n.a. [32]	n.a. [32]	up [32]
miR-1	n.a. [32], down [37, 38]	up [34]	up [32, 37], down [35]
miR-139-5p	n.a. [32]	up [32]	n.a. [32]
miR-188	n.a. [32]	up [32]	up [32]
miR-192	n.a. [32]	up [32]	n.a. [32]
miR-212	n.a. [32]	up [32]	n.a. [32]
miR-532	n.a. [32]	up [32]	up [32]
miR-133a	up [34], down [37]	no data	up [37]
miR-208a	up [32, 34]	n.a. [32]	down [32]
miR-320	up [34], down [32]	down [32]	down [32]
miR-487b	up [32]	down [32]	n.a. [32]

I/R: ischemia/reperfusion; Ipre: ischemic preconditioning; Ipost: ischemic postconditioning; n.a.: not affected.
